# Brain Networks Underlying Eye’s Pupil Dynamics

**DOI:** 10.3389/fnins.2019.00965

**Published:** 2019-09-18

**Authors:** Mauro DiNuzzo, Daniele Mascali, Marta Moraschi, Giorgia Bussu, Laura Maugeri, Fabio Mangini, Michela Fratini, Federico Giove

**Affiliations:** ^1^Fondazione Santa Lucia (IRCCS), Rome, Italy; ^2^Centro Fermi – Museo Storico della Fisica e Centro Studi e Ricerche Enrico Fermi, Rome, Italy; ^3^Donders Institute for Brain, Cognition and Behavior, Radboud University, Nijmegen, Netherlands; ^4^CNR Nanotec, Rome, Italy

**Keywords:** steady-state BOLD-fMRI, pupillometry, human brain, locus coeruleus, granger-causality, functional connectivity

## Abstract

Phasic changes in eye’s pupil diameter have been repeatedly observed during cognitive, emotional and behavioral activity in mammals. Although pupil diameter is known to be associated with noradrenergic firing in the pontine Locus Coeruleus (LC), thus far the causal chain coupling spontaneous pupil dynamics to specific cortical brain networks remains unknown. In the present study, we acquired steady-state blood oxygenation level-dependent (BOLD) functional magnetic resonance imaging (fMRI) data combined with eye-tracking pupillometry from fifteen healthy subjects that were trained to maintain a constant attentional load. Regression analysis revealed widespread visual and sensorimotor BOLD-fMRI deactivations correlated with pupil diameter. Furthermore, we found BOLD-fMRI activations correlated with pupil diameter change rate within a set of brain regions known to be implicated in selective attention, salience, error-detection and decision-making. These regions included LC, thalamus, posterior cingulate cortex (PCC), dorsal anterior cingulate and paracingulate cortex (dACC/PaCC), orbitofrontal cortex (OFC), and right anterior insular cortex (rAIC). Granger-causality analysis performed on these regions yielded a complex pattern of interdependence, wherein LC and pupil dynamics were far apart in the network and separated by several cortical stages. Functional connectivity (FC) analysis revealed the ubiquitous presence of the superior frontal gyrus (SFG) in the networks identified by the brain regions correlated to the pupil diameter change rate. No significant correlations were observed between pupil dynamics, regional activation and behavioral performance. Based on the involved brain regions, we speculate that pupil dynamics reflects brain processing implicated in changes between self- and environment-directed awareness.

## Introduction

Consensual changes in eye’s pupil size are strongly associated with neuromodulatory tone. Specifically, pupil size is controlled by sympathetic and parasympathetic systems (McDougal and Gamlin, 2015) and it is recognized as a peripheral index of arousal (Bradley et al., 2008; Gilzenrat et al., 2010; Jepma and Nieuwenhuis, 2011; Nassar et al., 2012; Wierda et al., 2012; Naber et al., 2013; Urai et al., 2017; Wang et al., 2018; Aminihajibashi et al., 2019; van Kempen et al., 2019), in addition to the well-known regulation by ambient illumination (reviewed by Mathot and Van der Stigchel, 2015). In monkeys and rodents, light-independent pupil dilations have been found to be correlated with neuronal firing in the pontine locus coeruleus (LC), the major source of noradrenergic input to the cerebral cortex (Rajkowski et al., 1993; Joshi et al., 2016). Accordingly, pupil dilations are associated with desynchronized cortical electroencephalographic and electrophysiological signals and enhancement of sensory responses, i.e., attentive state (Reimer et al., 2014).

Although the results found in animal models might not directly translate to the human brain, the relationship between pupil dynamics and LC has been indirectly confirmed in humans using pupillometry and functional magnetic resonance imaging (fMRI) (Alnaes et al., 2014; Murphy et al., 2014; Yellin et al., 2015; Schneider et al., 2016; Elman et al., 2017). An important difference between animal and human studies is that the fMRI signal only indirectly reflects neuronal firing through the action of neurovascular coupling (Logothetis and Pfeuffer, 2004). However, recent experimental evidence in mice demonstrated that resting-state hemodynamics can be predicted from spontaneous neuronal activity using a convolution-based approach, indicating that hemodynamic fluctuations are indeed a low-pass filtered version of the underlying neuronal activity (Ma et al., 2016). Moreover, either electrode recordings within LC or aggregate neuronal calcium signaling in cortical axonal projections from LC (the latter having an intrinsically slow dynamics, e.g., several hundreds of ms), have been reported to be closely correlated to pupil size fluctuations (Joshi et al., 2016; Reimer et al., 2016). Accordingly, pupil dilations after activation of LC occur on a relatively slow time-scale (500–1000 ms) (Larsen and Waters, 2018). These findings strongly support the possibility to track pupil-related brain activity using neurovascular coupling through the fMRI blood oxygenation level-dependent (BOLD) signals, as evidenced by several experiments. In particular, LC activation was initially observed using the temporal derivatives of the canonical hemodynamic response function (HRF) (Murphy et al., 2014 and references therein). Subsequently, it was found that it is not pupil size *per se* but the change rate of pupil diameter to be correlated with the BOLD signals in LC and other subcortical and cortical areas during resting state (Schneider et al., 2016). This notion is consistent with electrophysiological measurements in mice showing that, during both quiet waking and locomotion, pupil size is correlated with the cholinergic system, while its time derivative is correlated with the noradrenergic LC (Reimer et al., 2016). It should be noted, however, that LC does not directly control pupil size, and the functional relevance of anatomical pathways between LC and oculomotor neurons such as preganglionic cells of the Edinger-Westphal midbrain nucleus is not definitely established (McDougal and Gamlin, 2015). Thus, it is important to remark that fMRI is not able to resolve these dependencies, especially the correlations between regional brain activity and the high-frequency components of pupil dynamics.

Remarkably, during stimulus-related protocols in humans, such as reward-anticipation (Schneider et al., 2018) or fear-learning (Leuchs et al., 2017), but also during mind-wandering resting-state (Schneider et al., 2016), pupil-related activations consistently implicated cortical areas of the salience-network, including anterior cingulate cortex (ACC) and anterior insular cortex (AIC) (Menon and Uddin, 2010). Furthermore, a large degree of overlap has been reported in pupil-related BOLD activation maps during either continuous rest-fixation or block-design visual-imagery experiments (Yellin et al., 2015). These observations raise the possibility that at least in part low-frequency pupil dynamics reflects task-independent fluctuations in cortical activity.

In the present study, we sought to characterize the pupil-related brain networks during a steady-state experimental protocol designed to achieve a continuous degree of attentional load. To this end, we recorded pupillometry and fMRI data along with response times from healthy human subjects exposed to a whole-field visual stimulation and either left or right hemifield-directed attention. We were particularly interested in the brain network comprising LC and thalamus (TH), two critical components of the ascending reticular activating system (ARAS). The ARAS is responsible for arousal and it is thought to subserve both self and environmental awareness (Yeo et al., 2013). The aims of this work were (i) to interrogate the pupil-related brain networks during a continuous attentive state, and (ii) to identify the underlying functional hierarchy between the involved brain areas by means of causality and connectivity analysis.

## Materials and Methods

### Participants

Fifteen healthy volunteers (age: 26 ± 5 years, mean ± SD; age range: 18–44 years; 5 females) participated in the study. The protocol of the study was approved by the Ethics Committee of Santa Lucia Foundation. All subjects gave written informed consent in accordance with the Declaration of Helsinki and with the European Union regulations.

### MR Acquisitions

MR data were acquired on a 3T scanner (Siemens, Magnetom Allegra, Erlangen, Germany) equipped with a standard birdcage coil. Functional images were collected with a multi-echo (Speck and Hennig, 1998) planar imaging sequence (TR = 3100 ms, TE1/TE2/TE3 = 16/39/63 ms, Flip Angle = 85°, voxel size = 3× 3 × 3.75 mm^3^, FOV = 192 × 192 mm^2^) lasting 12 min and 43 s for a total of 246 volumes (including 4 dummy scans). Two functional runs were acquired for each subject. Parallel imaging and partial k-space sampling were avoided by leveraging the high scanner gradient performances (rise time 100 μs to reach a maximum amplitude of 40 mT/m). Clinical scans were also acquired to comply with institutional guidelines and to exclude pathological conditions.

### Steady-State Attentional Task

**FIGURE 1:**
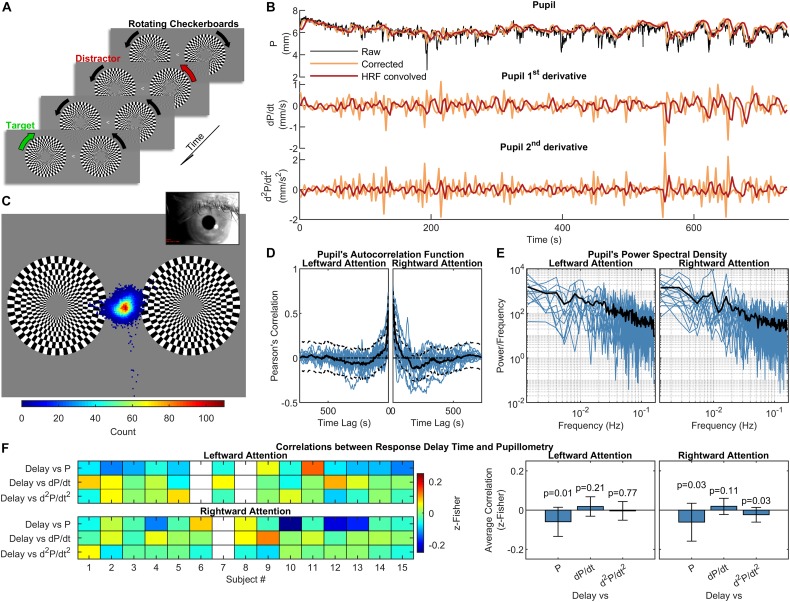
Experimental design, pupillometry and relation to subject’s performance. **(A)** Schematization of the visual stimulation with leftward attention presented during the functional scans. While maintaining the gaze on the central arrow, subjects had to pay attention to the change in the direction of rotation of the target checkerboard while ignoring the distracting checkerboard on the opposite hemifield. One run with leftward and one with rightward attention were acquired for each subject. **(B)** Pupil and pupil-derived time courses from one representative subject. **(C)** Eye-position heat map overlaid over the visual stimulation for one representative subject during the leftward attention run. The distribution is centered on the fixation point, demonstrating subject’s compliance. **(D)** and **(E)** panels show the autocorrelation function and power spectral density of the corrected and resampled pupil data, respectively. The black lines represent the across-subject averages, which are displayed over single-subject data (blue lines). **(F)** Correlations between response delay times and pupillometry over time, displayed separately for each subject (left panel) and for the entire group (right panel). The white rectangles on the left panel indicate missing data. The error bars on the right panel represent the standard deviation, while the *p*-values are the results of one-sample *t*-tests on the distribution of correlations.

During the acquisition of functional images, subjects performed a continuous motion detection task with covert attention. The constant-luminance (21 cd/m^2^) visual stimulation (Figure 1A) consisted of two circle-shaped (3° of radius) white-black rotating (2 cycles/s) checkerboards located in the left and right hemifield at 4° horizontal decentering and vertically centered. The two checkerboards inverted the direction of rotation independently of one another, with a random inversion period ranging uniformly from 1 to 3 s. A leftward or rightward white arrow located at the center of the screen identified the fixation point. Subjects were asked to keep the gaze on the central arrow while maintaining the attention to checkerboard pointed by the arrow, and to push an MRI compatible button whenever the target checkerboard inverted the direction of rotation. Two functional runs were acquired for each subject, with either leftward or rightward attention. The run ordering was randomized across subjects. The visual stimulation was generated with Cogent 2000 (Laboratory of Neurobiology, Wellcome Trust, London, United Kingdom) running on Matlab 7.1 (The Mathworks Inc., Natick, MA, United States). A single chip Digital Light Processing (DLP) projector model NP216G (NEC Display Solutions, Itasca, IL, United States), located outside the magnet room, projected the stimulation on a screen mounted on the magnet bore behind the subject, who viewed it via a mirror mounted on the head coil. The fixation distance between subjects’ eyes and the screen was approximately 65 cm.

### Preprocessing of Functional Images

Preprocessing of functional images was performed with AFNI (Cox, 1996). After discarding four dummy scans, correction for head motion was estimated from the second-echo series, using the first volume as reference (3dvolreg). Each echo series was slice-timing corrected (3dTshift) and then motion corrected by applying the previously estimated transformations (3dAllineate). Non-brain regions were removed by intersecting the realigned volumes with a mask obtained via 3dAutomask applied to a provisional optimal combined time series. Then, the three-echo series were fed to “tedana.py” to perform multi-echo ICA denoising (ME-ICA) (Kundu et al., 2012; Kundu et al., 2013). ME-ICA removes nuisance sources of variance by performing ICA decomposition followed by component classification based on their TE dependency, with TE-independent components marked as non-BOLD components and hence removed from the data. Tedana yielded a single cleaned “optimal combined” series, obtained via a T2^∗^-weighted average of the three echoes (Posse et al., 1999). The cleaned series was normalized to Montreal Neurological Institute (MNI) space (3 × 3 × 3 mm^3^) using as source image the mean of the motion-corrected second-echo series. The transformation to MNI space was obtained combining an affine transformation (@auto_tlrc) with a non-linear warp (3dQwarp), and it was applied in a single interpolation step using 3dNwarpApply. Slow frequencies (<0.008 Hz), linear and quadratic trends, as well as motion-derived parameters were regressed out using 3dTproject. Finally, the resulting series was smoothed within the brain mask with an isotropic 4 mm FWHM Gaussian kernel (3dBlurInMask).

### Pupillometry and Pupil Data Preprocessing

Eye-tracking data were acquired during complete darkness (ambient luminance measured inside the bore during the stimulation < 1 lux) in order to counter-balance the influence of the visual stimulation (at constant light intensity, see above) on pupil constriction, as addition of light facilitates the detection of spontaneous (i.e., light-independent) changes in pupil diameter (Behrends et al., 2019). Adaptation to the light conditions (ambient darkness plus visual stimulation) lasted ∼15 min while the acquisition of anatomical reference images and clinical scans took place. We did not test different light conditions, as eye-tracking measurements (e.g., number and duration of blink/eye closure) and fMRI-related variables (e.g., motion parameters and regional pupil-BOLD correlations) have been previously shown not to differ significantly in either light or dark conditions (Yellin et al., 2015; Schneider et al., 2016), although luminance might affect pupillary responses under cognitive load (Peysakhovich et al., 2017). During acquisitions, subject’s gaze was recorded (60 Hz) through an eye-tracking system (Applied Science Laboratories, model 504) equipped with remote pan/tilt optic infrared module and a video camera that was custom-adapted for use in the scanner. After a 16-points calibration procedure, eye-position traces, linearly interpolated and smoothed (after an up-sampling to 1 kHz) by a boxcar filter (width ± 25 ms), were used to verify subject’s compliance. Pupil diameter was directly computed by the eye-tracking software through automatic elliptical fitting and major axis determination (preferred over area to avoid partial pupil coverage by the eyelid). Peak-envelope of pupil diameter time course was determined based on its frequency-spectrum and downsampled to 1/TR to be used for regression analysis. Data underwent outliers-removal using Hampel Filter and detrending (only for linear trends), thus obtaining artifact-free pupil diameter time courses (Figure 1B). It is noted that the convolution approach used for pupil-BOLD regression carries the Nyquist frequency of 0.16 Hz, which means that our analysis of pupillary oscillations is correspondingly limited to low frequencies.

### Identification of Brain Regions Correlated to Pupil Dynamics

We performed a regression analysis by convolving the canonical HRF with the pupil diameter time-course as well as its first-order (pupil diameter change rate) and second-order time derivatives. The analysis was performed with SPM12^1^ running on Matlab 2018a (The Mathworks Inc., Natick, MA, United States). Statistical t-maps were thresholded at *p* < 0.001; Family-wise error (FWE) correction for multiple comparisons was performed at cluster level (*p*_FWE_ < 0.05). Each cluster identified a specific region of interest (ROI) used for further analysis (see below), with the following exceptions. Left and right orbitofrontal cortices, as well as anterior paracingulate cortex and cingulate cortex, were grouped together due to high homology and substantial temporal correlation between the corresponding time-series, thus reducing the eight clusters to six ROIs.

### Granger Causality Analysis

Pairwise conditional GC was computed using the state-of-the-art Multivariate Granger Causality (MVGC) Matlab Toolbox (Barnett and Seth, 2014). In particular, the algorithm performs numerical computation and statistical inference of multivariate GC given an ensemble of fMRI data time series (variables are the ROIs defined above, observations are time points, and trials are subjects/sessions). Since we found no difference in activation maps for left and right hemifield-directed attention, we lumped together all sessions. Vector autoregressive model parameter estimation was performed using the Levinson-Durbin-Whittle-Wiggins-Robinson (LWR) method and model selection was done using the Bayesian information criterion (BIC). Significance level was set to *p* < 0.05 with Bonferroni correction for multiple comparisons. The binarized version of the matrix of suprathreshold elements was interpreted as adjacency matrix for directed graph determination and calculation of node centrality.

### Seed-to-Voxel Functional Connectivity

With the aim of exploring the steady-state networks associated with the above-mentioned ROIs, we computed the seed-to-voxel functional connectivity (FC) maps (Biswal et al., 1995) as the Pearson’s correlation coefficient between the ROI-averaged time course and every other voxel time course. Group-level maps were generated with SPM12 using one-sample *t*-tests on the z-Fisher-transformed maps. FWE correction for multiple comparisons was performed both at voxel (*p*_FWE_ < 0.05) and cluster (*p*_FWE_ < 0.001) level.

### Data Availability

All data sets (fMRI-BOLD and pupil size time series) are available upon reasonable request to the corresponding author.

## Results

Pupillometry and steady-state fMRI data were successfully acquired from all subjects (two sessions from two different subjects were discarded due to low-quality of eye-tracking recordings and one session of a separate subject was discarded due to excessive in-scanner head motion). Overall task performance was high, with an average fraction of correct responses of 89 ± 10% (mean ± SD) and an average response delay time of 433 ± 42 ms (mean ± SD). All subjects kept their gaze consistently on the fixation point at the center of the screen (Figure 1C). As previously reported (Yellin et al., 2015), autocorrelation and frequency analysis revealed no periodic frequencies in the pupil data (Figures 1D,E). No significant correlation was found between mean performance and the average of either pupil diameter or its 1st- and 2nd-order time derivatives. A very small (*r* = *−*0.06) but significant (*p* < 0.05, one-sample, two-tailed t-test) negative correlation was observed using the response delay and the pupil diameter across time (Figure 1F).

**FIGURE 2:**
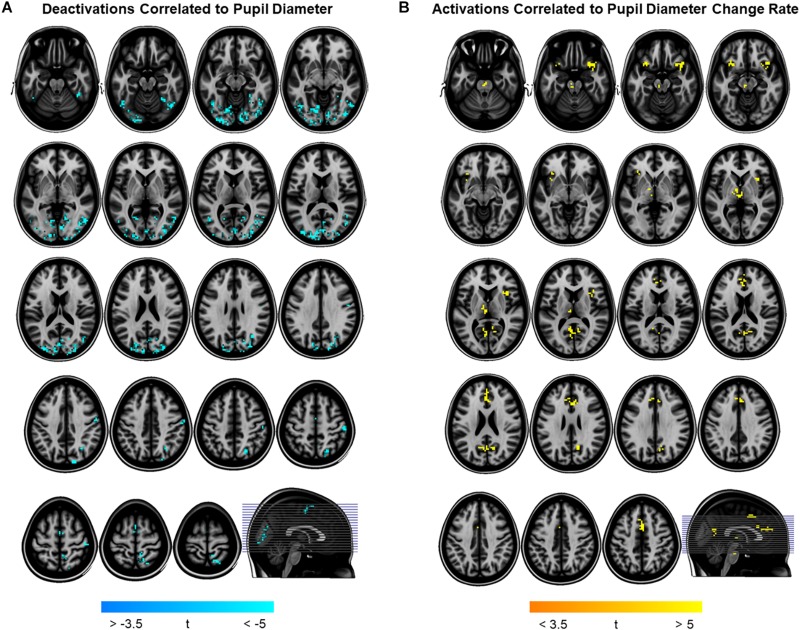
Brain regions correlated to pupil dynamics. Regression analysis yielded clusters of brain regions significantly (cluster-level *p*_FWE_ < 0.05) related to pupil diameter **(A)** and to pupil diameter change rate **(B)**, while no significant cluster was found testing the effect of pupil second-order time derivative. **(A)** Deactivations (i.e., negative correlations) were found by testing the effect of pupil diameter (see Table 1 for details). **(B)** Activations (i.e., positive correlations) were found by testing the effect of pupil diameter change rate (see Table 2 for details). Images are presented in neurological convention.

To determine pupil-related cortical areas, we performed regression analysis using general linear model by employing HRF-convolved pupil diameter and its time derivatives. Widespread deactivation maps correlated to pupil diameter were observed in visual and sensory-motor cortices (Figure 2A and Table 1). Activation maps correlated to pupil diameter change rate were observed in several subcortical and cortical areas (Figure 2B and Table 2), including LC, TH, posterior cingulate cortex (PCC), dorsal anterior cingulate and paracingulate cortex (dACC/PaCC), orbitofrontal cortex (OFC) as well as right anterior insular cortex (rAIC). Notably, separate regression analysis for left and right hemifield-directed attention revealed no suprathreshold cluster for either left > right or right > left statistical tests (*p* > 0.9 uncorrected, data not shown).

**TABLE 1:** Brain regions negatively correlated to pupil diameter.

**Region**	**Cluster**		**Voxel**			
	***p*_FWE_**	***k***	***t*_PEAK_**	***x***	***y***	***z***
Occipital fusiform/lingual gyrus (OF/LG) (R)	<0.001	38	7.94	15	−66	−6
Occipital fusiform/lingual gyrus (OF/LG) (L)	<0.001	114	6.46	−18	−72	−9
Lateral occipital cortex (LOC) (R)	<0.001	985	9.67	54	−66	−6
Postcentral/supramarginal gyrus (PC/SMG) (R)	<0.001	34	7.10	54	−27	54
Superior parietal lobule (SPL) (R)	<0.001	51	6.62	12	−48	63

*Reported clusters survived cluster-level Family-Wise Error (FWE) correction for multiple comparisons (*p*_*FWE*_ < 0.05). Voxel-level significance level was set to *p* < 0.001. Cluster size (in voxels) is indicated by *k*, while *t*_*PEAK*_ stands for the *t*-value of the peak voxel. Coordinates (*x*, *y*, *z*) are expressed in mm in the MNI-space.*

**TABLE 2:** Brain regions positively correlated to pupil diameter change rate.

**Region**	**Cluster**		**Voxel**			
	***p*_FWE_**	***k***	***t*_PEAK_**	***x***	***y***	***z***
Locus coeruleus (LC)	<0.01	17	6.21	−6	−27	−18
Thalamus (TH)	<0.001	37	7.11	−6	−18	−3
Posterior cingulate cortex (PCC)	<0.001	79	6.47	−9	−63	21
Dorsal anterior cingulate cortex (dACC)	<0.001	76	5.50	0	30	21
Anterior paracingulate cortex (PaCC)	<0.001	26	6.94	0	9	45
Orbitofrontal Cortex (OFC) (L)	<0.001	37	5.64	−30	18	−18
Orbitofrontal Cortex (OFC) (R)	<0.001	45	9.59	36	18	−18
Anterior Insular Cortex (AIC) (R)	<0.003	21	6.20	36	12	6

*Reported clusters survived cluster-level Family-Wise Error (FWE) correction for multiple comparisons (*p*_*FWE*_ < 0.05). Voxel-level significance level was set to *p* < 0.001. Cluster size (in voxels) is indicated by *k*, while *t*_*PEAK*_ stands for the *t*-value of the peak voxel. Coordinates (*x*, *y*, *z*) are expressed in mm in the MNI-space.*

**FIGURE 3:**
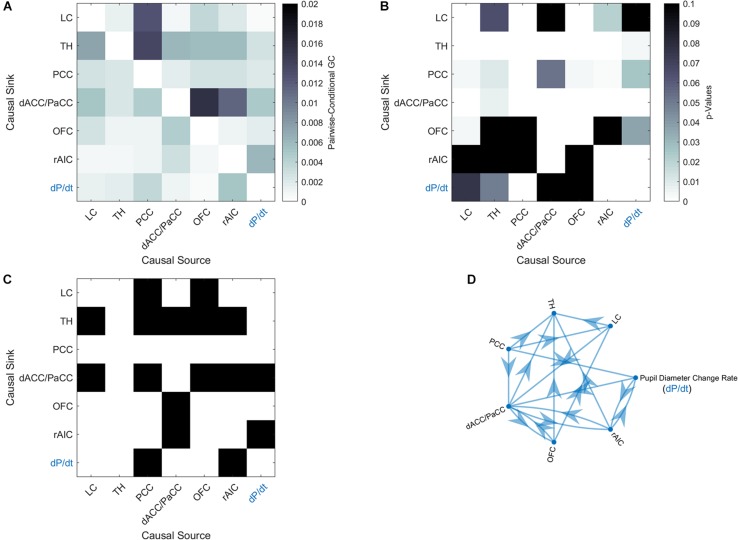
Granger-causality analysis for the pupil-related network. **(A)** Estimated pairwise conditional GC between BOLD time courses within each ROI. **(B)** Corresponding *p*-values. **(C)** Statistically significant (*p* < 0.05, Granger’s *F*-test, Bonferroni corrected) causal influences against the null hypothesis of no causality. **(D)** Directed graph of causal influences obtained using the result shown on panel C as adjacency matrix.

In order to elucidate the ability of each ROI (see section “Materials and Methods” for ROI definition) in predicting the behavior of the others within the pupil-related network, we performed Granger causality (GC) analysis. We found a complex pattern of interdependence between the cortical areas correlated with pupil diameter change (Figure 3). Specifically, PCC and dACC/PaCC exhibited the largest pairwise conditional GC for outwardly and inwardly directed causal influences, respectively. Node centrality analysis accordingly revealed that dACC/PaCC is the main authority and PCC is the main hub of the cortical network (Table 3). No significant correlation was found between mean within ROI *t*-value and either pupil dynamics or average task performance (*q*_FDR_ > 0.2, data not shown).

**TABLE 3:** Node centrality of ROIs within the pupil-related brain network.

**Name**	**Degree**		**Closeness**		**Betweenness**	**Pagerank**	**Hubs**	**Authorities**
	**in**	**out**	**in**	**out**				
LC	2	2	0.069	0.077	0	0.0946	0.1597	0.1462
TH	5	0	0.143	0	0	0.2445	0	0.3028
PCC	0	4	0	0.125	0	0.0511	0.2373	0
dACC/PaCC	5	3	0.139	0.099	11.0	0.2261	0.1083	0.2992
OFC	1	3	0.078	0.087	3.0	0.1152	0.1985	0.0364
rAIC	2	3	0.087	0.087	3.5	0.1609	0.1985	0.0691

*The different measures can be briefly described as follows. Degree: (in) number of incoming edges to each node, (out) number of outgoing edges from each node. Closeness: (in) normalized number of nodes reachable from it, (out) normalized number of nodes reaching it. Betweenness: regions most often found on the shortest path between two nodes. Pagerank: average time spent on each node during a random walk. Hubs: sum of the authorities scores of all its successors. Authorities: sum of the hubs scores of all its predecessors.*

**FIGURE 4:**
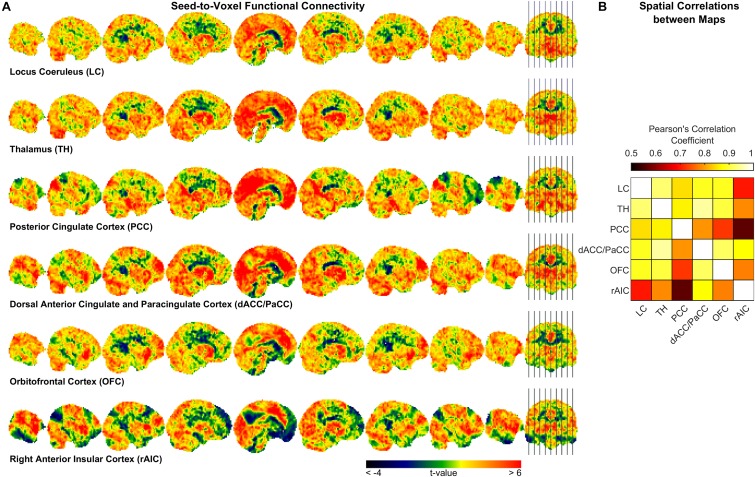
Seed-to-voxel functional connectivity analysis. **(A)** Unthresholded t-maps showing the second-level effect of seed-to-voxel connectivity (see Table 4 for the corrected statistics). The seed-ROI is indicated below each map. **(B)** Correlation matrix between each pair of ROI-seed FC maps.

To further characterize pupil-related networks, we determined seed-to-voxel FC maps with each ROI taken as seed (Figure 4A and Table 4). As expected, the PCC-seed FC yielded default mode network (DMN) areas including superior frontal gyrus (SFG). Subregions of the SFG were common members in all FC networks. The correlation between the different FC maps was globally very high, with PCC-seed versus rAIC-seed exhibiting the lowest correlation.

**TABLE 4:** Brain regions functionally connected to the ROIs of the pupil-related network.

**Region**	**Cluster**		**Voxel**			
	***p*_FWE_**	***k***	***t*_PEAK_**	***x***	***y***	***z***
*Locus coeruleus (LC)*						
Locus coeruleus	<0.001	76	27.33	6	–27	–15
Thalamus	<0.001	6	9.90	–6	–18	–6
Superior frontal gyrus (medial)	<0.001	6	9.85	–3	42	36
*Thalamus (TH)*						
Thalamus	<0.001	141	16.64	6	–18	0
Median cingulate and paracingulate cortex	<0.001	7	10.97	0	–21	27
Superior frontal gyrus (medial)	<0.001	6	10.64	–6	33	33
Superior frontal gyrus (medial)	<0.001	6	9.86	6	42	39
Calcarine fissure and surrounding cortex	<0.001	5	9.54	0	–63	12
*Posterior cingulate cortex (PCC)*						
Posterior cingulate cortex	<0.001	758	22.81	–9	–63	21
Angular gyrus (L)	<0.001	73	19.21	–42	–75	33
Angular gyrus (R)	<0.001	15	11.53	57	–66	21
Ventromedial prefrontal cortex	<0.001	80	14.78	–6	39	–27
Superior frontal gyrus (medial)	<0.001	23	13.65	–3	60	36
Superior frontal gyrus (dorsolateral) (R)	<0.001	15	12.08	21	45	48
Superior frontal gyrus (orbital)	<0.001	13	12.60	–3	36	–6
*Dorsal Anterior Cingulate and Paracingulate Cortex (dACC/PaCC)*						
Anterior cingulate and paracingulate cortex	<0.001	217	15.05	9	30	30
Superior frontal gyrus (dorsolateral) (L)	<0.001	16	11.27	–30	54	21
Superior frontal gyrus (dorsolateral) (R)	<0.001	39	11.89	24	45	18
Superior frontal gyrus (posterior)	<0.001	21	10.61	0	9	48
Thalamus (L)	<0.001	15	11.11	–9	3	0
Thalamus (R)	<0.001	16	11.66	6	0	–3
Calcarine fissure and surrounding cortex	<0.001	15	10.31	0	–75	15
Caudate nucleus (L)	<0.001	13	11.26	–12	15	–12
Caudate nucleus (R)	<0.001	8	12.60	18	9	–15
Insula (L)	<0.001	24	11.09	–42	15	–9
*Orbitofrontal cortex (OFC)*						
Orbitofrontal cortex (L)	<0.001	93	15.74	–33	21	–15
Orbitofrontal cortex (R)	<0.001	79	13.90	30	24	–15
Anterior cingulate and paracingulate cortex	<0.001	64	13.80	–6	30	30
Superior frontal gyrus (dorsolateral) (R)	<0.001	12	9.87	30	57	18
*Right anterior insular cortex (rAIC)*						
Insula (L)	<0.001	42	11.89	–33	15	6
Insula (R)	<0.001	69	22.29	33	18	3
Superior frontal gyrus (posterior)	<0.001	20	10.65	3	15	51
Superior temporal gyrus (L)	<0.001	8	10.28	–63	3	–9
Superior temporal gyrus (R)	<0.001	9	10.22	63	6	–6
Temporal pole (L)	<0.001	12	10.11	–54	15	–12
Temporal pole (R)	<0.001	9	9.31	57	–9	6

*Voxel-level significance threshold was set at *p*_*FWE*_ < 0.05 (Family-Wise Error correction for multiple comparisons). Only clusters with *p*_*FWE*_ < 0.001 are shown. Cluster size (in voxels) is indicated by *k*, while *t*_*PEAK*_ stands for the *t*-value of the peak voxel. Coordinates (*x*, *y*, *z*) are expressed in mm in the MNI-space. Seed regions are indicated in italics.*

## Discussion

In this study, we investigated the brain networks related to temporal variations of the eye’s pupil. We were mainly interested in examining the connection between pupil dynamics and LC in the pons, which together with TH and other subcortical structures (e.g., catecholaminergic nuclei) are linked to the arousal/ARAS system (Yeo et al., 2013). The regression analysis between steady-state fMRI signals and pupil diameter showed deactivations in a widespread cortical territory comprising visual and sensorimotor areas (Figure 2A and Table 1), in excellent agreement with previous studies (Murphy et al., 2014; Yellin et al., 2015; Schneider et al., 2016). Since the cholinergic system is thought to be correlated to pupil diameter (Reimer et al., 2016), the negative activations that we report here might be explained by a decreased cholinergic input to visual and sensorimotor areas. However, in humans the cholinergic nucleus basalis of Meynert innervates predominantly the limbic system (Mesulam, 2004) while the patterns of cholinergic innervation in the visual cortex varies considerably across mammalian species (Gu, 2003). Previous reports interpreted these deactivations as reflecting the suppression of interoceptive processes (including mental imagery) during alertness-induced pupil dilations, possibly mediated by the cholinergic branch of the ARAS (Yellin et al., 2015; Schneider et al., 2016). Further research is required to elucidate the significance of these results.

Positive activations were found using pupil diameter change rate (i.e., first-order time derivative of the pupil size) as regressor, which identified a set of subcortical and cortical regions including LC and TH as well as PCC, ACC, OFC and AIC (Figure 2B and Table 2). These areas largely overlap with previously published results (see Table 5). It should be noted that differences between findings unrelated to specific experimental design (e.g., rest-fixation resting-state, block-design or steady-state task, and so on) might result from a variety of sources, including but not limited to preprocessing of pupillometry and fMRI data, physiological noise (e.g., respiratory and cardiac rhythms) correction and statistical significance thresholds (e.g., voxel- and cluster-level correction for multiple comparisons).

**TABLE 5:** Summary of findings obtained in previous pupillometry-fMRI experiments.

**Study Ref.**	**Sample**	**Protocol (^∗^)**	**Regressor used**	**Anatomical locations (^∗∗^)**
Murphy et al., 2014	*n* = 14 Healthy subjects Age: 29 ±8 years (Mean ± SD) Age range: 21–48 years 6 Females	Rest-fixation or continuous attentive task	Pupil diameter + HRF derivatives	*Positive correlations*: Occipital gyrus (inferior, middle, superior) Fusiform gyrus Lingual gyrus Cuneus Supplementary Motor area^1^ Anterior cingulate cortex Inferior frontal gyrus^2^ Insular cortex Thalamus Midbrain Pons (LC) Medulla
Yellin et al., 2015	*n* = 20 Healthy subjects Age: 29 ± n.d. years (Mean ± SD) Age range: 26–48 years 13 Females	Rest-fixation or block-design visual imagery	Pupil diameter	*Positive correlations*: Inferior parietal lobule^3^ Posterior cingulate cortex Precuneus medial prefrontal cortex *Negative correlations*: Early visual cortices^4^ Central sulcus^5^ Lateral sulcus^6^
Schneider et al., 2016	*n* = 32 Healthy subjects Age: 26 ± 4 years (Mean ± SD) Age range: 18–35 years 17 Females Subjects were asked to refrain from caffeine and underwent a 2 h mild sleep deprivation protocol on the day of the experiment	Rest-fixation (dark versus light condition)	Pupil diameter	*Positive correlations*: Cerebellum Thalamus Caudate Nucleus Putamen *Negative correlations*: Occipital gyrus (inferior, middle, superior) Fusiform gyrus Lingual gyrus Cuneus Precentral gyrus^7^ Postcentral gyrus^8^ Supplementary motor area^1^ Superior temporal gyrus Temporal pole precuneus Insular cortex Parahippocampal gyrus Amygdala
			1st-order time derivative of pupil diameter	*Positive correlations*: Inferior frontal gyrus^2^ Middle frontal gyrus Superior frontal gyrus Anterior cingulate cortex Middle Cingulate Cortex Inferior parietal lobule^3^ Middle temporal gyrus Precuneus Insular cortex Thalamus Caudate Nucleus Putamen Brainstem Cerebellum *Negative correlations*: Occipital gyrus (inferior, middle, superior) Fusiform gyrus Lingual gyrus Cuneus Precentral gyrus^7^ Postcentral gyrus^8^ Paracentral gyrus Supplementary motor area^1^ Precuneus Parahippocampal gyrus Amygdala
			2nd-order time derivative of pupil diameter	*Positive correlations*: Middle temporal gyrus Middle cingulate gyrus Supramarginal gyrus Precuneus Thalamus *Negative correlations*: Occipital gyrus (inferior, middle, superior) Fusiform gyrus Lingual gyrus Cuneus Precentral gyrus^7^ Postcentral gyrus^8^

*(^∗^) All the listed experimental protocols within individual studies yielded almost identical results. (^∗∗^) The original nomenclature used in the relevant study is retained (see numbered footnotes for details). ^1^Posterior subregion of the superior frontal gyrus; ^2^Includes orbitofrontal cortex; ^3^Includes supramarginal gyrus and angular gyrus; ^4^Includes occipital gyrus (inferior, middle, superior), fusiform gyrus and lingual gyrus; ^5^Includes primary motor cortex and primary somatosensory cortex; ^6^Includes inferior frontal gyrus, insular cortex and temporal pole; ^7^Primary motor cortex; and ^8^Primary somatosensory cortex.*

The network of brain areas related to pupil diameter change rate was found to have PCC as its main cortical hub, as evidenced by GC analysis (Figure 3) and node centrality (Table 3). PCC appears to be the principal cortical region in initiating the communication with both LC and pupil. In turn, the edges connecting LC to pupil diameter change rate always involve dACC/PaCC and rAIC, the latter mediating the sole mutual connection with pupil. These findings are consistent with the hypothesis that LC is “informed” by the cortex (here directly by OFC and PCC and indirectly by ACC), which largely support the Aston-Jones and Cohen (2005) model. It is noted that the edges between the nodes of the network do not necessarily entail the presence of a direct connection or the absence of an indirect connection, as in our GC analysis not all brain regions are incorporated and even those that are incorporated as ROIs might be subregions of the actual labeled area. As an illustration, the TH cluster resulting from our analysis only comprises parts of specific thalamic nuclei (i.e., medial dorsal nucleus, ventral anterior nucleus, central lateral nucleus, and lateral posterior nucleus) and the absence of outward edges from TH cannot be interpreted as lack of thalamocortical connections.

In order to somewhat reduce such limitation, we performed seed-to-voxel FC. Interestingly, all cortical regions that were correlated with the pupil diameter change rate had the SFG (orbital, medial, dorsolateral or posterior subregion) within the corresponding FC network (Table 4), consistently with the proposed parcellation and connectivity patterns of this area (Li et al., 2013). It is noted that the hub of the network related to the pupil diameter change rate, namely PCC, coincides with the main hub of the DMN. The DMN is the seat of internally generated and self-directed brain activity (Stawarczyk et al., 2011). SFG is part of the DMN (Buckner et al., 2008) and it has been linked to self-awareness (Goldberg et al., 2006).

Consistent with our expectation that pupil dynamics reflects spontaneous, task-independent fluctuations in cortical activity, we did not find any significant correlation between pupil diameter (or its time derivatives), pupil-related brain regions and task performance. This is particularly interesting considering that the task required a constant and relatively high degree of attention, which is known to implicate the ARAS and related cortical areas. While it is possible that the correlation between pupil and performance depend on specific kind of attentive processes (e.g., top-down attention versus decision-making, see van Kempen et al., 2019), our results are consistent with electroencephalography-pupillometry data showing no correlation between pupil and reaction time (Hong et al., 2014). Generally, understanding of the conditions where a correlation between pupil and performance exists is not straightforward (van den Brink et al., 2016; Trani and Verhaeghen, 2018). Notably, LC activity and pupil does not necessarily correlate with behavioral performance (Varazzani et al., 2015). We only report a very small correlation between instantaneous response delay time and pupil diameter time course, which might be explained by the known relation between pupil dilation and increase in attentional load (Lisi et al., 2015).

Virtually all regions of the pupil-related network are known to be implicated in arousal, selective attention, salience, error-detection, decision-making and perception (Schneider et al., 2016), i.e., more generally, in consciousness. Pupil dynamics has been proposed to reflect both conscious (Kang and Wheatley, 2015) and preconscious (Laeng et al., 2012) brain activity. In particular, pupillometry might be an index of spontaneous fluctuations in the arousal state that are characterized by a switch between internally directed (e.g., mind wandering) and environmentally directed (e.g., on-task) attentional resources (Unsworth and Robison, 2018). Pupil size has also been linked to the subjective passage of time (Suzuki et al., 2016). Notably, time-perception tasks implicate the AIC (see Gili et al., 2018). Timing has been related to the metastability of cortical networks, something that is hypothesized to be dynamically “adjusted” by the PCC (Leech and Sharp, 2014). We found that the rAIC-seed FC network and the PCC-seed FC network exhibited the lowest between-map correlation (Figure 4B), although both rAIC and PCC were strongly correlated with the pupil diameter change rate (Figure 2 and Table 2). Notably, the within-ROI BOLD time course of rAIC and PCC were anticorrelated (*p* = 0.0029, one-sample, two-tailed *t*-test). These regions may subserve complementary functions, as evidenced by the finding that activation of rAIC can cause deactivation of the PCC in response to unexpected salient events (Sridharan et al., 2008). It has been proposed that the anterior insula signals the need to direct attention externally, thereby leading the PCC to reduce whole-brain metastability in order to facilitate the activation of cortical areas/networks that are appropriate for the current behavioral state (Leech and Sharp, 2014). Based on our results, we speculate that the eye’s pupil reflects continuous switches between self- (PCC) and environment- (OFC and rAIC) directed awareness (Sridharan et al., 2008; Scheibner et al., 2017).

It should be realized that cortical regions participate in the maintenance of conscious state through consciousness-entangled contributions that are region-specific. For example, OFC is implicated in metacognitive processes related to self-awareness (Lak et al., 2014; Xie et al., 2018), in synergy with the LC noradrenergic system (Sadacca et al., 2017). The right anterior insula is involved in interoceptive body representation (i.e., integration of autonomic, visceral and sensory information) and together with anterior cingulate and prefrontal cortex constitutes a task-independent network known to be linked to goal-directed behavior (Eckert et al., 2009 and references therein). Even the rAIC is related to the awareness of visceral and sensory information (Kotani et al., 2009). The PCC is part of a functional noradrenergic arousal circuitry (together with LC and TH, among others) and has been proposed as a cortical hub for consciousness (see section “Discussion” in Song et al., 2017).

## Conclusion

In conclusion, we used steady-state fMRI to study the relation between pupil dynamics and brain networks. Our results advance a potential important link between changes in pupil and cortical state (Schwalm and Rosales Jubal, 2017). It would be interesting to study how the pupil-related network evolves during altered states of consciousness. For example, resting-state fMRI during pharmacological modulation of arousal established the brainstem (putatively LC) and TH as two main subcortical structures involved in consciousness (Gili et al., 2013; Song et al., 2017). In our analysis, LC and TH had the SFG as a functionally connected cortical region, in common with all pupil-related cortical areas. This result advances the possibility that FC with SFG might be used as an index of the pupillometry-fMRI relation. Finally, disruption of pupillary responses has been linked to neurodegeneration such as Parkinson’s disease (Wang et al., 2016) and Alzheimer’s disease (Granholm et al., 2017). For example, the identification of pupil-related network and the study of its integrity might be useful as an early marker of cognitive decline, e.g., in MCI patients (Elman et al., 2017). Although the interpretation of the data is made difficult by the complexity of the interplay between pupil size regulation and brain state, our results provide useful information that might help designing new experiments aiming at further investigating the pupil-BOLD relation in the human brain.

## Data Availability

The datasets generated for this study are available on request to the corresponding author.

## Ethics Statement

The protocol of the study was approved by the Ethics Committee of Santa Lucia Foundation. All subjects gave written informed consent in accordance with the Declaration of Helsinki and with the European Union regulations.

## Author Contributions

MD conceived the idea. FG designed and supervised the experiments. GB, DM, MM, and FG performed the experiments. MD and DM analyzed the data and prepared the figures. MD wrote the manuscript. All authors contributed to the interpretation of the results, critically reviewed the manuscript, and approved the content of the manuscript.

## Conflict of Interest Statement

The authors declare that the research was conducted in the absence of any commercial or financial relationships that could be construed as a potential conflict of interest.

## Footnotes

1 http://www.fil.ion.ucl.ac.uk/spm
